# 

**Published:** 1987-11

**Authors:** 


### Contents

93 **A national doctor morbidity survey**
Huw Griffith
95 **General practice and hospital medicine—a new relationship**
Michael Whitfield
97 **A predictive index for postoperative deep vein thrombosis in thoracic surgery patients**
R. B. Richardson, C. Dufresne, G. E. Staddon, F. A. Chudry, P. Whitehead, K. Jeyasingham
101 **A case of dysphagia**
P. G. P. Stoddart, S. Holl
103 **Toboggan or not toboggan**
M. J. Cobby, E. A. Thomas, A. H. Troughton
104 **Wishful thinking**
J. L. Burton
105 **From our correspondents**
107, 111 **Letters to the Editor**
108 **British Society of Gastro-enterology, Bristol Meeting**
112 **Wessex Branch of Association of Clinical Pathologists, Bath**
114 **South West Orthopaedic Club, Bath**
116 **Marjorie Budd Lecture, Abdominal aortic aneurysms, Trials and tribulations, Sir Geoffrey Slaney**
117 **Index**
95, 96, 100, 102 **Book Reviews**

